# Offspring genetic diversity regulates rearing experiences that predict differential susceptibility to *Chd8* haploinsufficiency

**DOI:** 10.21203/rs.3.rs-6058389/v1

**Published:** 2025-03-03

**Authors:** Manal Tabbaa, Alexis Gamez, A’Di Dust, Maja Mataric, Pat Levitt

**Affiliations:** 1 Division of Neurology, Department of Pediatrics and Developmental Neuroscience and Neurogenetics Program, Children’s Hospital Los Angeles, The Saban Research Institute, Los Angeles, CA 90027, USA; 2 Keck School of Medicine of the University of Southern California, Los Angeles, CA 90033, USA; 4 Department of Computer Science, University of Southern California, Los Angeles, CA 90027, USA

**Keywords:** *CHD8* haploinsufficiency, maternal care, offspring behavior, genetic diversity, genetic reference panel, Collaborative Cross, trait heterogeneity, early life experience, rearing, autism, neurodevelopment, neurodevelopmental disorders

## Abstract

Mouse models of human disease focus on determining the direct impact of genetic mutations on phenotypes related to clinical presentations. For example, loss of function mutations in the autism-associated *CHD8* gene is highly penetrant for trait and behavioral abnormalities in children, but there is substantial clinical heterogeneity in the occurrence and extent of disruptions between individuals. Using a large genetic reference panel of mice, we recently showed that genetic background strongly regulates variability in trait disruptions caused by *Chd8* haploinsufficiency. Here, we hypothesized that genetics could also impact the variability in response to early life experiences, thus contributing to differential susceptibility to neurodevelopmental disorders. To examine how genetic diversity impacts rearing experience, we systematically observed the behavior of genetically diverse offspring raised by genetically identical mothers. The results reveal strain differences in pup and maternal behaviors. Machine learning analysis reveals that early life litter experiences are strong predictors of sex-dependent postweaning social, anxiety-like, and cognitive trait disruptions due to *Chd8* haploinsufficiency. The study suggests that offspring phenotypes in mutant models of disease are due to a combination of heritable and early experience factors, demonstrating the utility of incorporating genetic diversity in studies to model the mechanisms that underlie the heterogeneity of disrupted phenotypes in neurodevelopmental disorders.

## Introduction:

*CHD8* haploinsufficiency is highly penetrant for an autism spectrum disorder (ASD) diagnosis, intellectual disability disorder (IDD), and macrocephaly, along with other, less penetrant co-occurring symptoms^1–3^. There is, however, heterogeneity between impacted individuals in the occurrence and severity of highly penetrant trait and co-occurring trait disruptions due to *Chd8* haploinsufficiency. There is a knowledge gap in understanding the mechanisms that contribute to individual differences in the occurrence and severity of trait disruptions due to *CHD8* haploinsufficiency. Understanding the mechanisms underlying varying phenotypes in *CHD8* haploinsufficiency and other neurodevelopmental disorders (NDDs) is important to improve clinicians’ ability to support impacted individuals. Research in humans has indicated that genetic background contributes to a phenotypic spectrum of trait disruptions in ASD^4,5^. Likewise, we and others have demonstrated the impact of controlled variation of genetic background on brain structure and function by using genetic reference panels (GRPs). We recently leveraged genetically diverse, recombinant inbred GRP mouse strains with *Chd8* haploinsufficiency and found heterogeneity in the occurrence and severity of trait disruptions across strains that replicated the heterogeneity observed in clinical populations^6^. Therefore, evidence points to the genetic background as one factor that mediates heterogenous outcomes in *CHD8* haploinsufficiency. There is little understanding, however, of the early postnatal interaction of genes with the environment that contribute to variable phenotypic outcomes due to differences in response to experiences during early life development.

In rodent models of early life variation, systematically varying resources, such as limiting bedding and nesting, impact the frequency and patterns of dam provisioning, including attending to offspring and leaving the nest^7–9^. This variability in dam nest exits impacts the physical growth as well as metabolic, brain and behavioral development of the offspring^9–14^. Naturally existing variability in rat dams licking and grooming and arched-back nursing of offspring contributes to the brain and behavioral development of the offspring stress response^15^. Moreover, the presence of additional caregivers, including the father, impact offspring early life experiences, adult traits, and brain neurochemical systems^16–19^. Differences in the genetic background and behaviors of offspring can also regulate maternal care through indirect genetic effects, which can then impact offspring outcomes^20–23^. However, the significance of early life experiences in neurodevelopmental disorders is typically studied in single inbred strains, failing to replicate heterogeneity in the early social environment that exists in genetically diverse populations. While understudied from a genetic perspective, evidence from human and animal studies shows that variation in parental-offspring interactions is critical for contributions to lifelong expression of offspring biological and behavioral phenotypes.

Here, we aimed to determine the extent of variation in the preweaning litter environment due to offspring genetic background in a model of *Chd8* haploinsufficiency, and the impact of that variation on *Chd8*-driven trait disruptions across strains. To determine if the genetic background of the offspring impacts maternal and pup behaviors, genetically identical B6 dams, heterozygous for *Chd8* (*Chd8*^+/−^), were observed for maternal behavior towards their WT and *Chd8*^+/−^ offspring from 16 genetically diverse and inbred mouse strains. Moreover, we measured the age at which developmental milestones were first observed across strains. Finally, we investigated if maternal and pup behaviors across a subset of offspring related to trait development as well as trait disruptions due to *Chd8* haploinsufficiency. Results demonstrate that genetic background regulates individual differences in pre-weaning experiences that are predictors of differential susceptibility to *Chd8* haploinsufficiency.

## Results:

### B6-*Chd8*^+/−^ dam litter compositions and behaviors vary across a population of genetically diverse offspring strains

**Figure 1:**
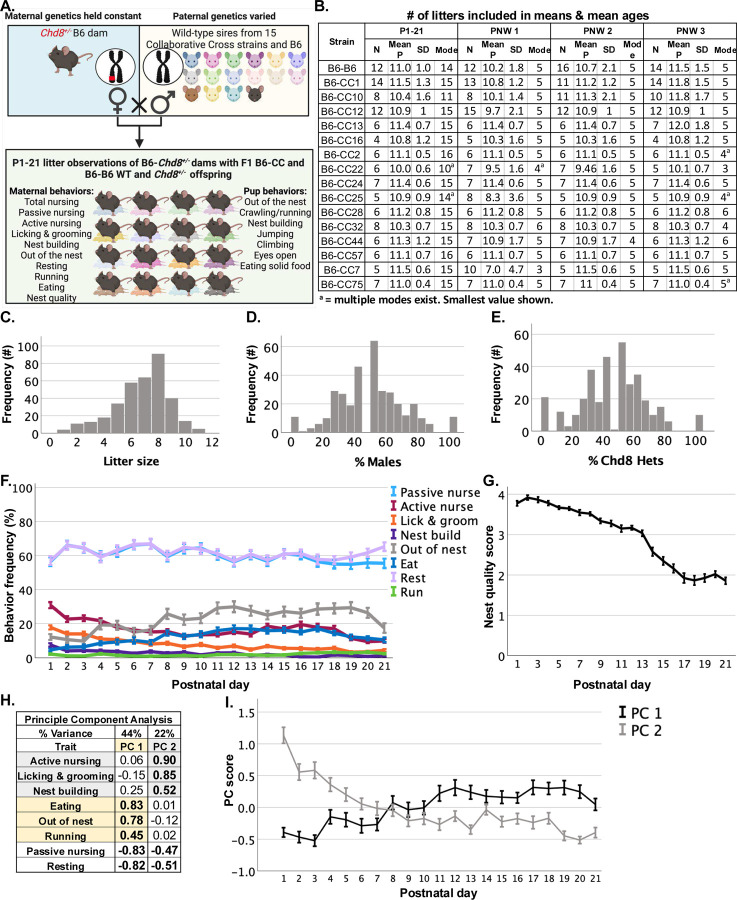
Observations of *B6-Chd8*^+/−^ dams genetically diverse litters: (A) B6 females that were heterozygous for *Chd8* (Chd8^+/−^) were paired with wild-type (WT) males from 15 Collaborative Cross (CC) strains in addition to B6 males to produce genetically diverse F1 B6-CC and B6-B6 WT and Chd8^+/−^ male and female progeny. Litter observations were conducted from postnatal day (P) 1 to weaning at P21 wherein instances of dam and pup behaviors were scored at 5-minute intervals over 1 hour for each litter. (B) The number of litters (N) that met inclusion criteria for each time bin analysis including during P1–21 and each postnatal week (PNW) as well as the mean postnatal day observed (Mean P) and standard deviation (SD). The mode for the number of postnatal days observed per litter (Mode P) is also listed. (C-E) Distributions of the number of pups and the percent of males and Chd8^+/−^ mice produced by B6- Chd8^+/−^ dams and CC and B6 sires (N=148 litters). (F) Percent behavioral frequencies of B6- Chd8^+/−^ dams across P1–21 in the combined offspring strain population. (G) Nest quality scores over postnatal development. (H) A principal component analysis with Varimax rotation and Kaiser Normalization revealed two principal components (PCs) that accounted for 68% of the total variance in B6- Chd8^+/−^ dam behavior. Bold text indicates dam variables that loaded highly (i.e., > .04) on each PC. PC1 and PC2 scores were calculated from respective loadings. (I) PC 1 increased in frequency over offspring postnatal development while PC 2 decreased over development, intersecting at P8. Line graphs show mean +/− standard error of the mean (SEM).

B6-*Chd8*^+/−^ females were mated with males from 15 Collaborative Cross (CC) strains and B6 to produce genetically diverse (*i.e.,* B6-CC) and identical (*i.e.,* B6-B6) F1 litters containing WT and *Chd8*^+/−^ offspring. Sires were separated from pregnant dams approximately 1 week prior to litter birth. Pregnant females were checked daily for litter births and the day of birth was designated as postnatal day (P) 0. From P1 until weaning at P21, litters were observed at approximately the same time for one hour per day at five-minute intervals during the light phase. During observations, the frequency of dam and pup behaviors were scored by a researcher blind to the strain of the offspring (Figure 1A). Prior to the start of each litter observation, nest quality was scored, following a previously described protocol^24^. The study design and the number of litters included in analyses per strain are illustrated in Figure 1A–B. The total number of observations per strain and postnatal day are listed in Supplementary Figure 1A. Multiple litters were included for each offspring strain over development (Figure 1B). B6-*Chd8*^+/−^ dams had genetically diverse litters that roughly followed a normal distribution in litter compositions, including litter size and the percentage of males and *Chd8*^+/−^ pups per litter (Figure 1C–E).

The longitudinal design of the study and large number of maternal trait measures allowed us to determine that the frequency of B6-*Chd8*^+/−^ dam behaviors towards the combined strain population of litters varied depending on the specific measure and postnatal day (Figure 1F). B6-*Chd8*^+/−^ dams spent most of the time resting and passively nursing during the light phase. Nest quality scores also varied over postnatal development with nest quality decreasing as offspring age increased (Figure 1G). The frequency of B6-*Chd8*^+/−^ dam behaviors observed across P1–21 were reduced statistically to factors that capture most of the behavioral variance via principal component analysis (PCA) (Figure 1H). Two PCs accounted for 66% of the total variance (Figure 4H). PC 1 represented dam-centered behaviors and included high loadings (*i.e.,* < 0.4) for out of the nest, running, and eating frequencies which were negatively related to passive nursing and resting. PC2 represented maternal care behaviors and included high loadings for active nursing, licking and grooming, and nest building, which were also more modestly negatively related to passive nursing and resting frequencies. Regression scores derived from the two PCs were calculated for each litter (Figure 1I). PC1 increased and PC2 decreased across pre-weaning development, intersecting and reversing directions in relation to each other by P8 (Figure 1I).

Litter size significantly correlated with multiple measures, with the largest correlation coefficients reflecting a negative association with nest quality scores (r = −0.52) and positive associations with dam out of the nest (r = 0.48) and eating (r = 0.48) frequencies (Supplementary Figure 1B). The percentage of males per litter was positively associated with nest quality scores with a small effect size (r= 0.22) while the percentage of *Chd8*^+/−^ pups per litter did not correlate with any measures (Supplementary Figure 1B).

Maternal and pup behaviors were significantly correlated, particularly activity behaviors during postnatal week (PNW) 3 (Supplementary Figure 1C). Dam out of the nest and eating behavior frequencies correlated with pup activity, including pup crawling/running, autogrooming, licking and grooming, eating solid food, digging, and eyes open, with mostly small to medium effect sizes. Dam out of the nest was positively correlated with pup autogrooming (R^2^ = 0.32) and pup eyes open (R^2^ = 0.25), with large effect sizes (Supplementary Figure 1C).

### Litter survival and dam behaviors across pre-weaning development vary depending on offspring strain

**Figure 2:**
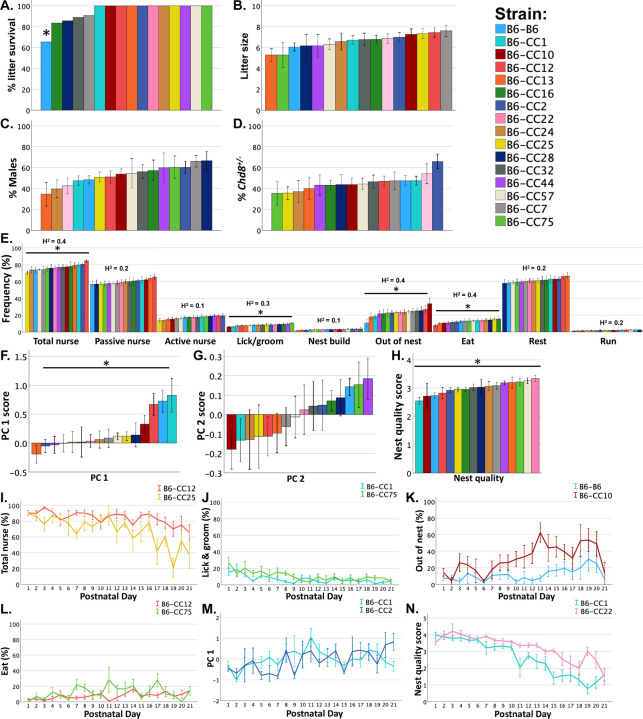
Offspring strain regulates litter survival, maternal behavioral frequencies, and nest quality across P1–21: (A) B6-Chd8^+/−^ dams that mated with B6 sires and gave birth to B6-B6 litters had fewer litters survive to weaning compared to B6-Chd8^+/−^ dams that mated with CC sires and birthed B6-CC litters. (B-D) There were no statistically significant differences in the litter size, percentage of males, and percentage of Chd8^+/−^ offspring in B6-Chd8^+/−^ dams litters across 16 offspring strain groups. (E) Offspring strain regulated the average percent frequency of B6-Chd8^+/−^ dam behaviors during 1-hour observations across postnatal days (P) 1–21 including total nursing, licking and grooming, out of the nest, and eating behaviors. H^2^ values represent broad-sense heritability estimates of B6-Chd8^+/−^ maternal behaviors which are the proportion of phenotypic variance in dam behavior accounted for by offspring strain compared with the total phenotypic variance. (F-H) Offspring strain also impacted B6-Chd8^+/−^ dam PC 1, but not PC 2 scores, and nest quality means across P1–21. (I-N) Line graphs show differences across P1–21 in B6-Chd8^+/−^ dam percent behavioral frequencies (I-L), PC 1 (M), and nest quality scores (N) between two extreme offspring strains. Bar and line graphs show mean +/− SEM and asterisks denote significant differences across offspring strain groups at p < .05 from LMMs with litter size, percentage of males and Chd8^+/−^ pups per litter, and dam parity covaried.

There was a significant effect of offspring strain on litter survival at weaning (X^2^ = 34.72, p < .01). Follow-up comparisons with Bonferroni corrected p values revealed that B6-*Chd8*^+/−^ dams that gave birth to B6-B6 litters had a lower proportion of litters that survived to weaning compared to all other strains (Figure 2A). Offspring strain did not significantly impact litter size, nor the percentage of males and the percentage of *Chd8*^+/−^ pups per litter (Figure 2B–D).

To determine if offspring strain impacts B6-*Chd8*^+/−^ dam behaviors, B6-*Chd8*^+/−^ dam trait frequencies across offspring strain groups were analyzed with Linear Mixed Models (LMMs) with litter size, the percentage of males and the percentage of *Chd8*^+/−^ pups per litter, and dam parity covaried to control for variance in dam behaviors due to these factors. B6-*Chd8*^+/−^ dam behaviors from P1–21 were analyzed for differences across offspring strain groups. Offspring strain impacted B6-*Chd8*^+/−^ dams total nursing (F_15, 99_= 2.53, *p* < 0.01), licking and grooming (F_15, 99_= 2.074, *p* < 0.05), out of the nest (F_15, 99_ = 1.918, *p* < 0.05), and eating (F_15, 99_ = 1.94, *p* < 0.05) frequencies (Figure 2E). B6-*Chd8*^+/−^ dams passive nursing, active nursing, nest building, resting, and running mean frequencies did not differ as a function of offspring strain during the preweaning period. Furthermore, B6-*Chd8*^+/−^ dams PC 1 scores differed across offspring strains (F_15, 99_ = 2.966, *p* < 0.001; Figure 2F) but not PC 2 scores (Figure 2G). Offspring strain also impacted nest quality score means (F_15, 90_= 3.412, *p* < 0.001; Figure 2H).

The heritability of B6-Chd8^+/−^ maternal behaviors was estimated as the proportion of phenotypic variance in dam behaviors explained by offspring strain, relative to the total phenotypic variance (Figure 2E). Among these behaviors, total nursing, licking and grooming, out of the nest, and eating frequencies showed moderate heritability (H^2^ > 0.2), while all other behaviors exhibited low heritability (H^2^ < 0.2),

Bonferroni corrected post-hoc comparisons were conducted to determine which offspring strain groups differed from each other on measures that showed significant differences and are fully detailed in Supplemental Table 1, with effect size estimates highlighting the magnitude of strain differences. Figure 2I–N shows examples of B6-*Chd8*^+/−^ dam behaviors and nest quality scores for strains that differed from each other with large effect sizes. B6-*Chd8*^+/−^ dams with B6-CC12 offspring had more total nursing frequencies compared to dams with B6-CC25 (Figure 2I; d = 2.57) offspring. In addition, B6-*Chd8*^+/−^ dams with B6-CC1 offspring were observed to lick and groom less frequently compared to dams with B6-CC75 offspring (Figure 2J; d = 1.47). B6-*Chd8*^+/−^ dams with B6-CC10 offspring were also observed to leave the nest more frequently compared to dams with B6-B6 offspring (Figure 2K; d= 1.38). B6-*Chd8*^+/−^ dams with B6-CC12 offspring were observed to eat less frequently than dams with B6-CC75 offspring (Figure 2L; **d = 0.90**). In addition, B6-*Chd8*^+/−^ dams with B6-CC1 offspring had higher PC 1 scores compared to dams with B6-CC2 offspring (Figure 2M; d = 1). Lastly, litters with B6-CC1 offspring had reduced nest quality scores compared to litters with B6-CC22 offspring (Figure 2N; d = 2.56). These data demonstrate that offspring strain impacts B6-*Chd8*^+/−^ maternal care and nest quality across pre-weaning development, with large effect sizes.

**Figure 3:**
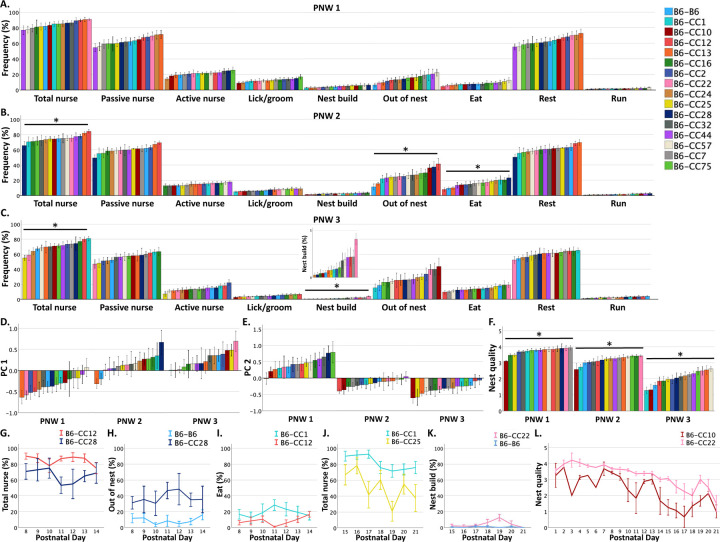
Offspring strain regulates B6-*Chd8*^+/−^ dam maternal behavioral frequencies and nest quality across specific postnatal weeks: (A) During postnatal week (PNW) 1, B6-*Chd8*^+/−^ dam percent behavior frequencies did not differ as a function of offspring strain groups. (B) During PNW 2, B6-*Chd8*^+/−^ dam total nursing, out of nest, and eating frequencies differed across offspring strains. (C) During PNW 3, B6-*Chd8*^+/−^ dam total nursing, and nest building frequencies differed across offspring strains. (D) B6-*Chd8*^+/−^ dams PC 1 and PC 2 scores did not differ across offspring strain groups at PNW 1, PNW 2, and PNW 3. (F) Nest quality scores significantly differed as a function of offspring strain during PNW 1, PNW 2, and PNW 3. (G-L) Line graphs highlight differences in dam behaviors across postnatal days for minimum and maximum offspring strains during PNW 2 (G-I) and PNW 3 (J-K) and nest quality scores across P1–21 (L). Mean +/− standard error of the mean (SEM) is graphed and asterisks denote significant differences across offspring strain groups at p < .05 from LMMs with litter size, percentage of males and Chd8^+/−^ pups per litter, and dam parity covaried.

Because the combination of dam behaviors changes over time prior to weaning, and offspring undergo biological and behavioral changes across specific developmental epochs, we next binned the data into separate postnatal weeks (PNWs). There were no significant differences in B6-*Chd8*^+/−^ dam’s behavioral frequencies observed across offspring strains groups during PNW 1 (Figure 3A). There were also no differences in PC means across offspring strains during PNWs 1, 2, and 3 (Figure 3D–E). During PNW 2, B6-*Chd8*^+/−^ dams’ total nursing (F_15, 105_ = 1.926, *p* < 0.05), out of nest (F_15, 105_ = 3.109, *p* < 0.001), and eating (F_15, 105_ = 2.188, *p* < 0.05) frequencies differed as a function of offspring strain (Figure 3B). During PNW 3, total nursing (F_15, 104_ = 2.982, *p* < 0.001) and nest building (F_15, 104_ = 2.805, *p* < 0.001) frequencies differed across offspring strains (Figure 3C). Nest quality scores differed across offspring strains across each of the weeks (PNW 1: F_15, 96_ = 2.228, *p* < 0.05; PNW 2: F_15, 87_ = 3.183, *p* < 0.001; PNW 3: F_15, 92_ = 5.553, *p* < 0.001; Figure 3F). This indicates that nest quality scores are sensitive to offspring strain during all PNWs while the behavior of B6-*Chd8*^+/−^ dams is more sensitive to offspring strain during later PNWs. The data suggest time-dependent differences of the influence of genetic background on specific dam behaviors.

Post-hoc comparisons, detailed in Supplementary Table 1, identified the strains that differed from each other across each PNW, including effect sizes. Examples are highlighted in Figure 3G–L. During PNW 2, B6-*Chd8*^+/−^ dams with B6-CC12 offspring had increased total nursing frequencies compared to dams with B6 CC28 offspring (d = 1.97; Figure 3G). In addition, B6-*Chd8*^+/−^ dams with B6-B6 offspring exited the nest less frequently during PNW 2 than dams with B6-CC28 offspring (d = 1.58; Figure 3H). During PNW 2, B6-*Chd8*^+/−^ dams with B6-CC12 offspring were observed to eat less frequently than dams with B6-CC1 offspring (Figure 3I; d = 0.9).

During PNW 3, B6-CC25 litters had decreased total nursing compared to B6-CC1 litters (Figure 3J; d = 2.4). During PNW 3, dams with B6-CC22 offspring had increased nest building activity compared to B6-CC10 offspring (d = 3.21; Figure 3K).

Reduced nest quality scores throughout PNWs 1–3 were driven by a few strains with large effect sizes, including B6-CC10 (Figure 3L), B6-CC1, and B6-B6 litters having reduced nest quality scores compared to the majority of the other strain groups (Supplementary Table 1).

**Figure 4:**
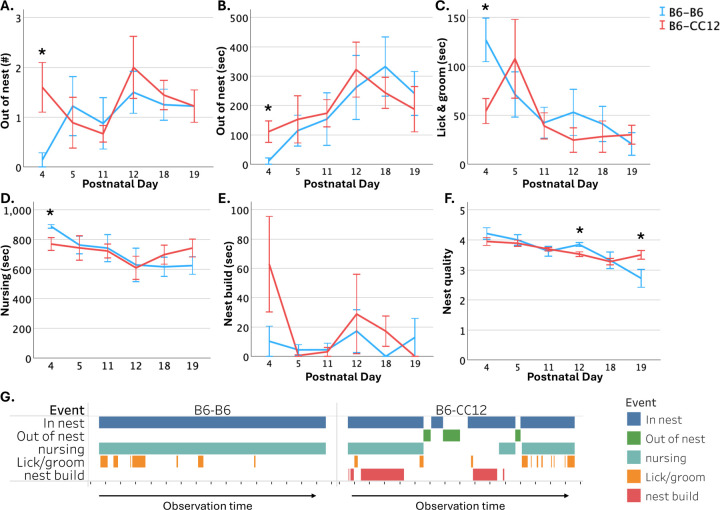
B6-*Chd8*^+/−^ dam maternal behavioral frequencies and durations differ across two offspring strain groups: (A-B) B6-*Chd8*^+/−^ dams that had B6-CC12 offspring exited the nest more frequently and for increased durations at P4 compared to dams with B6-B6 offspring. (C-D) B6-*Chd8*^+/−^ dams with B6-CC12 offspring spent less time licking and grooming pups and nursing, compared to dams with B6-B6 offspring. (E) There were no significant differences in B6-*Chd8*^+/−^ dams nest building durations between offspring strains. (F) Nest quality scores differed between B6-CC12 and B6-B6 litters at P12 and P19. (G) Representative examples of activity of B6-*Chd8*^+/−^ dams with B6-B6 and B6-CC12 litters during 3 5-minute behavioral observations performed on P4. Each tick mark on the x-axis represents one minute. Mean +/− standard error of the mean (SEM) is graphed. * = p < 0.05 with litter size, percentage of males and *Chd8*^+/−^ pups per litter, and dam parity covaried.

Next, to understand if durations and patterns of B6-*Chd8*^+/−^ dam’s behavior differ as a function of offspring strain, the frequency and duration of maternal behaviors of B6-*Chd8*^+/−^ dams with B6-B6 offspring and B6-CC12 offspring were quantified and compared at select days representing the 3 week period preweaning (P4, P5, P11, P12, P18, and P19; N = 11–12 litters per strain). There was substantial variation in the timing of expressed differences in the frequency and duration measures of maternal behavior. B6-*Chd8*^+/−^ dams with B6-CC12 offspring had increased out of nest frequencies (F_1, 15_ = 5.579, *p* < 0.05; d = 1.12), and durations (F_1, 15_ = 5.128, *p* < 0.05; d = 1.1) compared to dams with B6-B6 offspring on P4 (Figure 4A–B). In addition, B6-*Chd8*^+/−^ dams with B6-CC12 offspring had decreased licking and grooming (F_1, 15_ = 4.874, *p* < 0.05; d = 1.5) and nursing (F_1, 15_ = 5.138, *p* < 0.05; d = 1.1) durations on P4, compared to dams with B6-B6 offspring (Figure 4C–D). There were no significant differences in the time B6-*Chd8*^+/−^ dams with B6-CC12 versus B6-B6 offspring spent nest building, but B6-B6 litters had increased nest quality scores on P12 compared to B6-CC12 litters (F_1, 15_ = 8.412, *p* < 0.05; d = 1.5) while B6-CC12 litters had increased nest quality scores on P19 (F_1, 16_ = 16.882, *p* < 0.001; d = 1.1), compared to dams with B6-B6 offspring (Figure 4E–F). B6-*Chd8*^+/−^ dam maternal care towards representative litters with B6-B6 versus B6-CC12 offspring on P4 is illustrated in Figure 4G. These data further show the complexity of B6-*Chd8*^+/−^ dams behavior, with differences that are influenced by offspring strain and developmental time.

### Genetic background impacts litter behaviors

**Figure 5:**
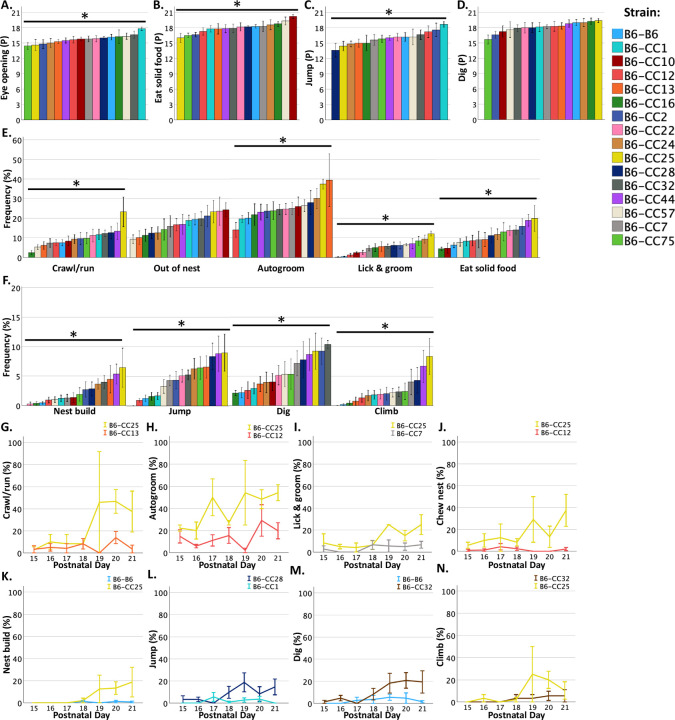
Genetic background regulates developmental milestones and litter behaviors: (A-D) The mean age at which behaviors were first observed in each litter significantly differed across 16 strain groups including age at eye opening (A), eating solid food (B), and jumping (C), but not digging (D). (E-F) The frequency of behaviors observed in at least one pup during litter observations averaged across postnatal week 3 differed across strains including crawling/running, autogrooming, licking and grooming, eating solid food, nest building, jumping, digging, and climbing, but not out of the nest. (G-O) Line graphs highlight differences in litter behavioral frequencies across postnatal days during PNW 3 from extreme strains. Mean +/− standard error of the mean (SEM) is graphed. * = p < 0.05 with litter size, percentage of males and Chd8^+/−^ pups per litter, and dam parity covaried.

Most pup behaviors that were included in the inventory first emerged during PNWs 2 and 3 (Supplementary Figure 2). The average age that litters were first observed to display developmental milestones including eye opening, eating solid food, jumping, and digging were analyzed for strain differences. Not all litters displayed all the behaviors and only litters that displayed each behavior were included in analyses for age differences across strains. The number of litters excluded in the analysis of strain differences in developmental milestones is listed in Supplementary Figure 3. There were not enough litters for all strains that displayed climbing behavior and therefore climbing behavior was not analyzed for differences in mean ages when climbing was first observed. The average age of eye opening (F_15, 93_ = 2.823, *p* < 0.001; Figure 5A), eating solid food (F_15, 84_= 3.137, *p* < 0.001; Figure 5B) and jumping (F_14, 65_ = 2.171, *p* < 0.05; Figure 5C) differed across strains. Interestingly, B6-CC10 pups did not display jumping and climbing during pre-weaning development. There were no strain differences in the age at first observed to dig (Figure 5D).

Next, the mean frequency of pup behaviors during PNW 3 was analyzed for strain differences. During PNW 3, strain significantly impacted pup crawling/running frequencies (F_15, 92_ = 2.879, *p* < 0.01), autogrooming (F_15, 92_ = 3.181, *p* < 0.001), licking and grooming (F_15, 92_ = 8.408, *p* < 0.001), eating solid food (F_15, 92_ = 2.415, *p* < 0.01), nest building (F_15, 92_ = 2.557, *p* < 0.01), jumping (F_15, 92_ = 5.614, *p* < 0.01), climbing (F_15, 92_ = 3.45, *p* < 0.001), and digging (F_15, 92_ = 3.093, *p* < 0.001) frequencies (Figure 5E–F).

The results of Bonferroni corrected post-hoc comparisons across strains for significant differences in the mean percent frequencies of pup behaviors observed per litter are listed in Supplementary Table 2. Representative strain differences are graphed in Figure 5G–N and highlighted in the text below. Strain differences in eating frequencies did not survive Bonferroni corrections for post-hoc comparisons.

Litters with B6-CC1 pups took an average of 3 days longer before eye opening was first observed, compared to litters with B6-CC75, B6-CC13, and B6-CC24 pups (Supplementary Table 2). B6-CC10 offspring took an average of 4 days longer to be first observed to eat solid food compared to B6-CC25, B6-CC75, and B6-CC2 offspring (Supplementary Table 2). B6-CC1 offspring were first observed to jump on average 5 days later than offspring from the B6-CC28 strain Supplementary Table 2). These data indicate that genetic background impacts the age at which developmental milestones are first displayed.

Levels of activity also differed across strains during preweaning development. B6-CC25 pups were observed to crawl/run more frequently during PNW 3 compared to 63% of the other strains, with large effect sizes, including B6-CC13 (d = 1.51) in Figure 5G. B6-CC12 pups were observed to autogroom less frequently during PNW 3 compared to 19% of the other strains, with large effect sizes, including B6-CC25 pups (d = 2.40) in Figure 5H. B6-CC25 pups were more frequently observed to lick and groom siblings and/or the dam compared to 56% of the other strains, with large effect sizes, including B6-CC7 (d = 2.57) in Figure 5I. In addition, B6-B6 pups were observed to nest build less frequently during PNW 3 compared to B6-CC25 (d = 1.58; Figure 5K) pups. B6-CC13, B6-CC25, B6-CC28, B6-CC44, and B6-CC75 pups were observed to jump more frequently during PNW 3 compared to B6-B6, B6-CC1, B6-CC10, and B6-CC12 strains, with large effect sizes, including B6-CC28 versus B6-CC1 (d = 1.86) in Figure 5L. B6-CC32 pups displayed digging behavior more frequently during PNW 3 compared to B6-B6 (d = 2.95; Figure 5M). Lastly, B6-CC25 pups climbed more frequently during PNW 3 compared to 56% of the other strains, with large effect sizes, including B6-CC32 in Figure 5N (d = 1.33). These data implicate strain in regulating offspring developmental milestones, activity levels, and the pre-weaning litter environment.

### Variation in the preweaning environment predicts differential susceptibility to *Chd8* haploinsufficiency

**Figure 6:**
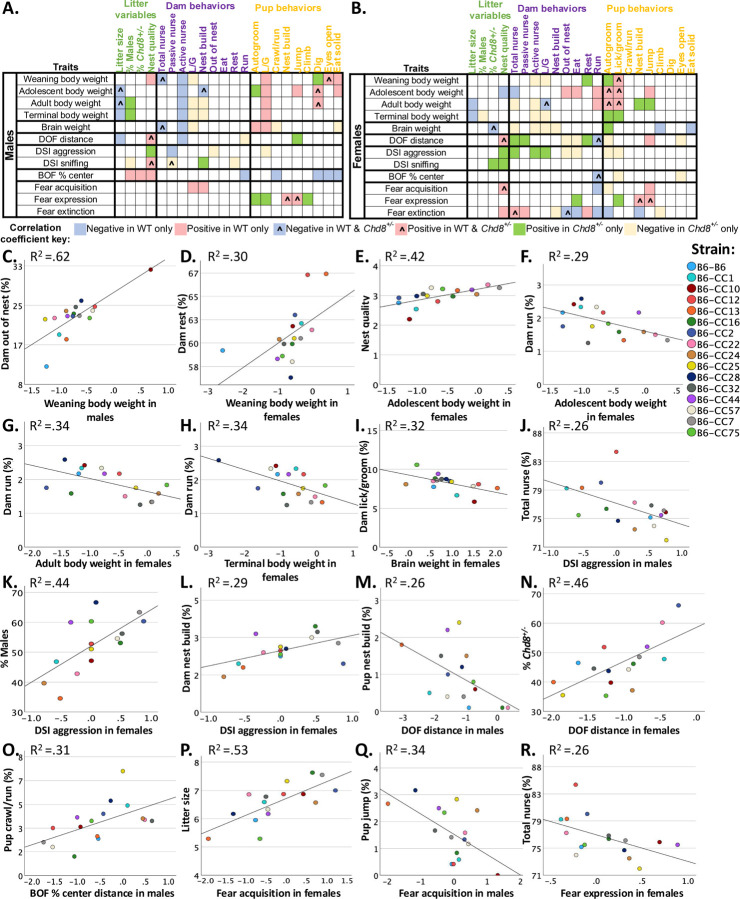
Variability in the preweaning environment is related to postweaning traits and *Chd8* effect sizes: (A, B) Spearman’s correlation coefficients identified preweaning litter observation variables and postweaning offspring traits that significantly correlated in WT and *Chd8*^+/−^ mice in the combined strain population with small-medium effect sizes. Only significant correlations are shown. Blue color represents a negative association in WT while red represents a positive association in WT. Blue and red squares with ^ symbol represent a negative and positive association in both WT and *Chd8*^+/−^ mice. Green reflects a positive association and yellow reflects a negative association in *Chd8*^+/−^ mice only. (C-D) Litter observation measures that significantly predicted strain *Chd8*^+/−^ effect sizes for trait disruptions in a sex dependent manner. Each dot represents the average litter measure and Cohen’s D effect size for one strain and sex group. R^2^ values reflect the coefficient of determination from linear regression.

A major goal of the present study is to determine how early life experiences relate to postweaning traits in WT and *Chd8*^+/−^ males and females. To accomplish this, postweaning traits for individual subjects in the combined strain population were correlated statistically with litter composition, nest quality, and the average frequencies for dam and pup behaviors that were observed across P1–21 (Figure 6A–B). Postweaning traits included in analyses are listed in Figure 6A/B, and we have previously reported the impact of strain on these traits^6^. The results indicate that litter composition measures, dam behaviors, and pup behaviors, significantly correlate with postweaning traits differently in WT and *Chd8*^+/−^ males and females, with mostly small (r=.1–.29) to medium (r=.3–.49) effect sizes^25^. In the text, we highlight differences in correlations between WT and *Chd8*^+/−^ males and females that were further validated as significant predictors with univariate linear regression (statistics reported in Supplementary Table 3).

Dam licking and grooming of pups was negatively correlated with and predicted weaning and terminal body and brain weights in *Chd8*^+/−^, but not WT, males and females, with small effect sizes. Thus, *Chd8*^+/−^ males and females’ body and brain weight development may be more sensitive to dam licking and grooming compared to WT.

The percentage of males in each litter was predictive of terminal body weights in *Chd8*^+/−^, but not WT, males and females, with small effect sizes and a positive correlation in *Chd8*^+/−^ males and a negative correlation in *Chd8*^+/−^ females. Moreover, the percentage of *Chd8*^+/−^ siblings per litter was associated with increased sniffing in the DSI task in *Chd8*^+/−^ females, with a small effect size. Interestingly, the percentage of *Chd8*^+/−^ littermates was also positively correlated with and predictive of the percentage of distance traveled in the center of the BOF test in WT males, with small effect sizes, suggesting that the *Chd8* litter composition preweaning can impact anxiety-like behavior in WT mice.

Dam out of nest frequencies were negatively correlated with DSI aggression in *Chd8*^+/−^ but not WT males and females, with small effect sizes. Dam out of nest instances were also negatively correlated with distance traveled in the DOF test in *Chd8*^+/−^, but not WT, females, with a medium effect size. Moreover, pup autogrooming was positively correlated with brain weight, and negatively correlated with distance traveled in the DOF test, with medium correlation effect sizes, and were significant, but weak, predictors in *Chd8*^+/−^, but not WT, females. In *Chd8*^+/−^ males, but not WT, pup crawl/run frequencies were negatively correlated and predictive of brain weight, with a small effect size. These data indicate that early life litter observation measures including litter compositions and dam behaviors are predictive of adult traits in the combined strain population differently between WT and *Chd8*^+/−^ subjects and depending on sex.

Next, to understand if preweaning litter observation measures predict differential susceptibility to altered postweaning traits due to *Chd8* haploinsufficiency, litter composition, nest quality scores, and the average frequencies for dam and pup behaviors that were observed across P1–21 were correlated with Cohen’s D effect sizes for trait disruptions across 16 strains (Supplementary Table 4). Univariate regression next tested if litter observation measures that were correlated with effect sizes also predicted differential susceptibility to trait disruptions due to *Chd8* haploinsufficiency (Figure 6C–R). Dependent variables tested included strain Cohen’s D effect sizes for each adult trait measured (N=8 subjects per sex and *Chd8* genotype).

Strain variability in decreased body weights in *Chd8*^+/−^ compared to WT was the trait outcome that was predicted by most of the preweaning litter observation measures. Specifically, dam leaving the nest predicted *Chd8*^+/−^ effect sizes for reduced weaning body weights in males across strains (F_1, 15_ = 22.665, p<.001); increased dam out of the nest was associated with decreased *Chd8*^+/−^ effect sizes (Figure 6C). In females, dam resting frequencies predicted strain effects of *Chd8*^+/−^ on reduced weaning body weights (F_1, 15_ = 5.853, p<.05) with increased dam rest frequencies associated with decreased effect sizes (Figure 6D). Dam running frequencies predicted *Chd8*^+/−^ effect sizes on female adolescence (F_1, 15_ = 5.633, p<.05; Figure 6F), adult (F_1, 15_ = 7.156, p<.05; Figure 6G), and terminal (F_1, 15_ = 7.060, p<.05; Figure 6H) body weights, with increased running frequencies associated with increased *Chd8*^+/−^ effect sizes. These data indicate that the dam may alter her behavioral strategy depending on the susceptibility of her offspring to *Chd8*^+/−^ induced body weight loss.

Nest quality also predicted *Chd8*^+/−^ strain effect sizes on female adolescent body weights (F_1, 15_ = 10.047, p<.01; Figure 6E) with increased nest quality associated with decreased *Chd8*^+/−^ effect sizes. Higher nest quality may thus be protective of *Chd8*^+/−^ driven adolescent body weight loss in females.

*Chd8*^+/−^ effect sizes across strains for macrocephaly (*i.e.,* increased brain weights) in females was predicted by dam licking and grooming frequencies, with increased dam licking and grooming associated with decreased effect sizes (F_1, 15_ = 6.527, p<.05; Figure 6I). The data suggests another protective role dam licking and grooming behavior could play on *Chd8*^+/−^ driven macrocephaly. *Chd8*^+/−^ effect sizes for increased DSI aggression durations in females were predicted by the percentage of male littermates, with increased aggression effect sizes associated with increased male littermates (F_1, 15_ = 11.063, p<.01; Figure 6K). *Chd8*^+/−^ effect sizes for increased DSI aggression durations in females was also predicted by frequencies of dam nest building (F_1, 15_ = 5.604, p<.05; Figure 6L), with increased DSI aggression effect sizes associated with increased dam nest building. The results suggest that in female *Chd8*^+/−^ mice, increased DSI aggression may be influenced by the percentage of male littermates and the frequency of dam nest building behaviors. Alternatively, increased aggression in *Chd8*^+/−^ mice may be driven by an unknown genetic factor that is regulating both increased male littermates and aggressive behavior. It also is possible that increased aggression in *Chd8*^+/−^ mice may be driving dam nest building behavior. In contrast, male *Chd8*^+/−^ mice exhibited a different pattern, where increased total nursing frequencies associated with decreased *Chd8*^+/−^ effect sizes for DSI aggression (F_1, 15_ = 4.842, p<.05; Figure 6J).

Interestingly, decreased *Chd8*^+/−^ effect sizes for reduced distance traveled in the DOF test in females was associated (F_1, 15_ = 12.027, p<.01; Figure 6N) with increased percentages of *Chd8*^+/−^ littermates suggesting a potential protective effect of increased genetic relatedness within the litter. In contrast, *Chd8*^+/−^ effect sizes for reduced DOF distance in males was predicted by pup nest build frequencies (F_1, 15_ = 5.028, p<.05; Figure 6M), with increased nest building associated with increased effect sizes.

Increased pup crawl/run frequencies predicted *Chd8*^+/−^ strain effect sizes for anxiety-like behavior in the BOF test in males (F_1, 15_ = 6.167, p<.05; Figure 6O), with increased crawl/run frequencies associated with decreased effect sizes. This suggests that pup crawl/run is an early indicator of *Chd8*-driven increased anxiety effect sizes in adult males.

*Chd8*^+/−^ effect sizes for fear-related behaviors in adulthood were predicted by litter size (fear acquisition: F_1, 15_ = 15.944, p<.01; Figure 6P) and total nursing frequencies (fear expression: F_1, 15_ = 4.85, p<.05; Figure 6R) in females. In males, *Chd8*^+/−^ effect sizes for fear acquisition were predicted by pup jump frequencies (F_1, 15_ = 7.184, p<.05; Figure 6Q). These results suggest that the impact of *Chd8*^+/−^ on fear-related behaviors may be modulated by environmental factors that are sex dependent.

Together, these results identify measures in the preweaning litter environment that predict *Chd8*^+/−^-driven outcomes in sex-specific ways and highlight the complex interplay between genetic, environmental, and preweaning environment in shaping *Chd8*^+/−^ driven outcomes.

**Figure 7:**
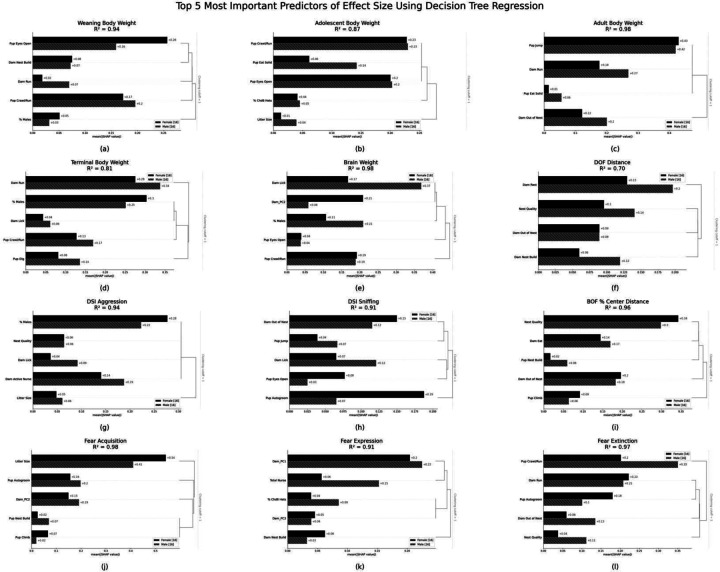
Preweaning litter observation measures are robust predictors of postweaning *Chd8*^+/−^ strain effect sizes across 12 trait outcomes: Results from decision tree regression. SHAP (SHapley Additive exPlanations) values quantify the relative contribution of each litter observation measure, averaged across strain, to the *Chd8*^+/−^ strain effect sizes for each outcome (A-L). SHAP values are represented on the x-axis and separated by sex. Females are represented by black bars, and males are represented by hatched bars. Predictors are represented on the y-axis and feature importance is indicated by the SHAP values on the x-axis and as also indicated by the number next to each bar, with higher SHAP values indicating greater variable importance. The dendogram to the right of each graph shows which features are most correlated to each other. The R^2^ value reflects the proportion of variance that is explained by the model.

Finally, to evaluate the potential impact of interactions among different litter observation measures on predicting *Chd8*^+/−^ strain effect sizes, and to identify the most important predictors of *Chd8*-driven phenotypic outcomes, we conducted a decision tree regression analysis. We then used SHAP (SHapley Additive exPlanations)^26^ interpretation to further understand the relationships in the decision tree models by quantifying the relative contribution of each litter observation measure to predicting *Chd8*^+/−^ strain effect sizes (Figure 7). This approach allowed us to assess both the strength of individual predictors and the interactions between multiple features, offering a comprehensive view of how different preweaning litter observation measures contribute to variability in *Chd8*^+/−^ strain effect sizes. The decision tree regression model was designed to capture non-linear and non-parametric relationships among the variables and their influence on trait outcomes. SHAP values interpret how individual measures influence the deviation from the expected value (average of the dependent variable) to the final predicted value, offering a transparent framework for interpreting a more complicated model such as a decision tree.

Results revealed that pre-weaning litter observation measures were consistently strong predictors of *Chd8*^+/−^ strain effect sizes, with their predictive power varying between male and female offspring. Notably, the contribution of preweaning litter observation measures to predicting phenotypic outcomes differed across sexes. Further, comparison between models using individual versus combined litter observation measures showed that integrating multiple litter observation measures outperformed models based on single measures alone (Figure 6C–6R vs Figure 7). These analyses suggest that the interaction between different litter observations adds a layer of predictive power that is not captured when considering these factors in isolation. The top predictors of *Chd8*^+/−^ strain effect sizes were dependent on the specific postweaning trait being measured. These predictors included both dam and pup behaviors. Additionally, for certain outcomes, litter composition measures, such as pup sex ratios or the number of pups per litter, were also found to be influential. The ranking of these predictors varied between males and females, suggesting that *Chd8*^+/−^-driven outcomes may be influenced by pre-weaning behaviors in a sex-dependent manner.

## Discussion:

Complex traits and their disruptions are regulated by complex interactions between genes and the environment. In genetically diverse populations, these factors include an individual’s genetic background (i.e., direct genetic effects) and the genetically influenced behaviors of other individuals in their environment (i.e., indirect genetic effects). Here, using a robust model of murine genetic diversity, we show that the genetic background of the offspring impacted specific features of maternal care as well as offspring developmental milestones and frequency of expressed behaviors. The comprehensive analysis revealed that, beyond the differences observed across the genetically diverse strains, the early life litter environment can predict both strain-specific variations in trait outcomes and disruptions caused by *Chd8* haploinsufficiency. This finding has important implications for modeling human neurodevelopmental disorders (NDDs). Most pre-clinical models of NDDs rely on studying trait alterations in a single inbred strain, which limits the study of gene X environment interactions, particularly during sensitive periods of development, and restricts the applicability of findings to genetically diverse human populations. By incorporating both genetic and environmental heterogeneity, preclinical models can more accurately reflect the complexity of NDD risk and uncover underlying mechanisms that may otherwise remain undetected in single-strain models. This approach can significantly enhance an understanding of NDDs and lead to more robust therapeutic strategies.

Here, we show that maternal behaviors are impacted as a function of offspring genotype. Notably, there were fewer numbers of B6-B6 litters that were born to B6-*Chd8*^+/−^ dams and survived to weaning compared to B6-CC litters. We, and others, have previously reported that pup survivability is reduced in litters that are born to B6-*Chd8*^+/−^ dams that breed with WT sires compared to WT dams that breed with B6-*Chd8*^+/−^ sires and rear WT and *Chd8*^+/−^ mixed genotype litters^23,27^. We now show that B6-*Chd8*^+/−^ dams with B6-B6 litters of mixed *Chd8* genotypes have fewer litters that survive to weaning compared to B6-*Chd8*^+/−^ dams with B6-CC litters of mixed *Chd8* genotypes. These data suggest that there is an interaction between *Chd8*^+/−^ in the dam and in the litter that impacts reduced survivability of the litter in the B6 background. We previously reported that maternal behavior of B6-*Chd8*^+/−^ dams is altered towards litters of the same genetic background, compared to B6-WT dams. In addition, B6-B6 pups may be particularly sensitive to this altered maternal care while B6-CC offspring may be more resilient to these alterations in dam behaviors^23^.

Total nursing, licking and grooming, out of the nest, and eating frequencies of B6-*Chd8*^+/−^ dams differed across offspring strains during P1–21. PC1 scores of dam-centered behaviors also differed. Temporal analysis revealed expected differences due to changes in maternal-offspring interactions that occur across preweaning development. Given the emergence of pup maturation over this period, we analyzed variation in measures by week, revealing that total nursing varied across offspring strains both during PNWs 2 and 3, whereas out of the nest and eating frequencies only varied as function of offspring strain during PNW 2. Total nursing frequencies were negatively correlated with weaning body weights during PNWs 2 and 3 in the combined strain population, suggesting that offspring body weight impacted the frequency of total nursing during later PNWs. Most differences in dam behaviors across offspring strain groups appeared during PNWs 2 and 3, but not PNW 1. The later periods include the onset of pup departures from the nest and an increase in motor activity suggesting that differences in dam behaviors may be due to offspring behaviors. Differences in dam behaviors across offspring strain groups may be due to several factors, including 1) variability in offspring traits due to genetic background (e.g., WT body weights), 2) differential susceptibility to trait disruptions due *Chd8* haploinsufficiency (e.g., body weight loss), and 3) WT and *Chd8*^+/−^ behaviors (e.g., increased WT locomotor activity or decreased *Chd8*^+/−^ activity^6^).

Comparisons of the frequency of behaviors displayed by pups during preweaning development revealed strain differences in almost all pup behaviors measured. In addition, the age at which developmental milestones were first observed varied across strain groups, including the age at eye opening, eating solid food, and jumping. Maternal and offspring interactions are reciprocal and correlated, and thus, we suggest that these differences in pup behaviors are likely to be influencing maternal behaviors, particularly during PNWs 2 and 3.

Nest quality scores also differed across offspring strains during PNWs 1, 2, and 3, while nest building behaviors by the dam differed across offspring strains during PNW 3. Nest quality is impacted by the activity of the pups, which varies across strains, and thus dams’ nest building behaviors may be sensitive to changes in late nest quality when pups become more active. Other factors besides pup activity may be regulating nest quality scores in earlier postnatal weeks. For example, during PNW 1, B6-CC10 litters, which display lower activity compared to other strains across P1–21, had significantly lower nest quality scores compared to B6-CC7 and B6-CC22 litters. During PNW1, nest quality is particularly critical for providing a source of insulation during development and thermoregulation critical for body weight gain^24^. Thus, another factor that may be regulating differences in nest quality scores, particularly during PNW 1, may be susceptibility to body weight loss due to *Chd8* haploinsufficiency. *Chd8* haploinsufficiency is generally associated with reduced body weights in mice, compared to WT. However, B6-CC10 *Chd8*^+/−^ males have been shown to be resilient to body weight loss at weaning and were the only strain and sex group out of 33 studied to show significantly *increased* body weights in adolescence compared to WT^6^. Therefore, we suggest that nest quality scores may be reduced in B6-CC10 litters, compared to other strains, due to B6-CC10 males’ unique metabolism that also protects them from body weight loss due to *Chd8* haploinsufficiency. Litters with B6-CC10 pups also were observed to eat solid food an average of 4 days later compared to 3 other strains. However, female B6-CC10 *Chd8*^+/−^ have been shown to have lower adolescent and adult body weights compared to WTs, and in this study, we found that nest quality scores predict strain effect sizes for adolescent body weights in females. The direction of the correlation suggests that increased nest quality scores is associated with reduced strain effect sizes for body weight loss in adolescent females. Therefore, nest quality score differences in B6-CC10 litters, compared to other strains, may be regulated by the differential needs of male and female offspring and unique metabolism of the B6-CC10 strain.

Variability in the frequency and duration of dam arched-back nursing, out of the nest, and licking and grooming behaviors have been shown to regulate brain development and behavior of the offspring in other rodent models^7–10,15^. Therefore, differences in out of the nest, licking and grooming, and total nursing may serve as additional factors that impact the outcomes of offspring in a model of *Chd8* haploinsufficiency. For example, we found that licking and grooming across P1–21 is predictive of macrocephaly effect sizes in adult female strains. When additional litter observation measures are considered, decision tree regression revealed that dam licking and grooming is also an important feature in predicting brain weight effect sizes in adult males. The negative correlation suggests that lower levels of licking and grooming by the dam across P1–21 is associated with larger effect sizes for macrocephaly across strains. This may occur by reducing resilience that develops through maternal-pup interactions. Moreover, we showed that whereas one behavior exhibits a predictive outcome correlation in one sex, a different behavior has a predictive outcome in the other sex. For example, total nursing frequency was predictive of DSI aggression strain effect sizes in males while the percentage of male littermates and dam nest building frequency were univariately predictive in females. In addition, machine learning modeling revealed that when the interaction between traits was considered, the significance of litter observation measures in predicting *Chd8*^+/−^ strain effect sizes differed across sexes. The data also suggest that early life experiences can impact both morphological and behavioral offspring trait alterations due to *Chd8* haploinsufficiency.

The present study design also allowed for investigating sibling interactions, which may be an additional variable that impacts development and adult outcomes. The percentage of male siblings positively correlated with DSI aggression strain effect sizes in adult *Chd8*^+/−^ females. Moreover, the percentage of *Chd8*^+/−^ siblings in the litter is expected to alter the rearing environment considering the impact *Chd8* haploinsufficiency has on regulating adult phenotypes. Indeed, we found that increased percentages of *Chd8*^+/−^ siblings in the litter predicts increased DSI sniffing in *Chd8*^+/−^ females in the combined strain population. Surprisingly, increased percentages of *Chd8*^+/−^ siblings in the litter also predicted decreased strain effect sizes for reduced locomotor activity in the DOF test in females. These data suggest that *Chd8*^+/−^ mice co-housed together can serve as a modulator of behavioral outcomes due to *Chd8* haploinsufficiency. Our findings align with a previous study using B6 mice that found that varying the ratio of co-housed *Chd8*^+/−^ mice for 4 weeks beginning at weaning impacted social and anxiety-like behaviors, brain activity, and the microbiome of *Chd8*^+/−^ mice^28^.

The present study measured dam and pup behaviors during the light phase of the diurnal cycle when mice are less active. Therefore, it is possible that differences across groups were minimized, in comparison to observations made during the dark phase, when mice are more active. It is important to understand differences in dam and pup behaviors as a function of genetic background across both the light and dark phase and future work will aim to include observations, using a reverse light:dark cycle, in order to fully understand how rearing experience may differ and contribute to outcomes in NDD models. Furthermore, our litter observations sampled snapshots of behavioral frequencies over one hour per day. The design, with a large number of collaborative cross strains that required observation across preweaning development, posed challenges that are associated with breeding, housing, and inclusion of both WT and *Chd8* haploinsufficient genotypes. We recognize that while the data collection strategy used was sufficient to capture differences in dam and pup behaviors with medium-large effect sizes as a function of offspring strain, differences in behaviors that were observed less frequently may be due to the sampling rate.

In summary, our results demonstrate that genetic diversity among offspring influences maternal provisioning, pup development, and preweaning experiences, with large effect sizes. Across genetically diverse strains, variability in specific dam, pup, and litter measures predicted *Chd8*^+/−^ effects. These findings emphasize the complex, multifactorial nature of trait variation and highlight the critical role of both maternal and litter dynamics in predicting outcomes driven by *Chd8* haploinsufficiency. By incorporating a range of behavioral and compositional factors, greater accuracy can be achieved for predicting phenotypic disruptions associated with *Chd8* mutations across diverse genetic backgrounds. Moving forward, it will be important to explore how these early rearing experiences impact adult phenotypes and susceptibility to specific gene mutations that lead to NDDs. Preclinical models that capture the heterogeneity of genetic and environmental risk factors for NDDs offer opportunities to model individual experiences and gain valuable insights into underlying mechanisms that will be overlooked in single-strain models. This approach could significantly enhance our understanding of NDDs and offers new avenues for personalized medicine and prevention strategies.

## Methods

### Animals:

C57BL/6J (B6) mice that were heterozygous for *Chd8* (*Chd8*^+/−^) were originally received from Dr. Feng Zhang (MIT). This B6-*Chd8* mouse line was generated through Cas9-mediated germline editing followed by germline transmission and inheritance. *Chd8*^+/−^ mice in this study are descendants from one founder with germline transmission of a loss-of-function *Chd8* allele containing a 7-nucleotide deletion in exon 1, resulting in a 50% reduction in CHD8 protein expression at embryonic day 18 compared to WT littermates^29^. B6-*Chd8*^+/−^ dams were bred with B6 males and males from 15 Collaborative Cross strains, all obtained from The Jackson Laboratory (Bar Harbor, ME), at 6–8 weeks of age and allowed to habituate to the colony for at least one week before breeding. CC strains were chosen based on health data provided for CC strains so that strains noted to have reduced survivability or health challenges were avoided. Notably, the sire was removed from the home cage prior to litter births to control for individual differences in paternal care. Breeding pairs were checked daily for signs of pregnancy and sires were removed from breeding cages approximately 1 week prior to litter births. All mice were weaned at P21 and genotyped for *Chd8* with validated in-house genotyping protocols using approximately 1–1.5 mm long tail snips collected at weaning. At euthanasia, additional tail samples were harvested, and subjects were genotyped for a second time to confirm *Chd8* genotype. Litter observations were conducted in three cohorts over 4 years. The first and second cohorts included 12 and 4 strains, respectively, and litter observations on these strains measured frequency behaviors across P1–21. Mice in cohorts one and two were housed in the Ray R. Irani vivarium at the University of Southern California (USC) main campus from 2019–2021. Cohort 3 consisted of 2 strains and litter observations on these strains measured frequency and duration behaviors during P4, P5, P11, P12, P18, and P18. Mice in cohort 2 were housed in The Saban Research Institute at Children’s Hospital Los Angeles (CHLA) in 2023. All mice were housed in standard ventilated cages on a 12 h light/dark cycle with lights on at 6:00 AM in a temperature (20–22° C) and humidity (40–60%) controlled room with ad libitum access to standard rodent chow and filtered water. All mice were weaned at P21 and housed with 2–5 same-sex cagemates. Litter size and the percentage of males and the percentage of *Chd8*^+/−^ littermates were recorded at weaning because handling of the pups pre-weaning adds an experimental variable that may impact dam behaviors differently across experimental groups. A subset of subjects was tested for traits in adulthood, including body weights at weaning, adolescence, adulthood, and at euthanasia (*i.e.,* terminal weights), dark open field (DOF) activity, direct social interaction (DSI), bright open field (BOF) activity, and fear conditioning. Brain weights were also measured for these subjects. Details regarding behavioral testing and results have been described in Tabbaa et al., 2023^6^. All experimental procedures were approved by the USC and CHLA Institutional Animal Care and Use Committees under protocols 11844-CR011 and 430. In addition, all experimental procedures followed the Guidelines for the Care and Use of Laboratory Animals by the National Institutes of health.

### Litter observations:

All litter observations were conducted in a quiet suite, adjacent to the main housing room, and dedicated to mouse behavior observations. At least one hour prior to the start of observations, cages with litters were transferred from the main colony to standard tables so that there was an unobstructed view of all cage sides by the research observer. The hour prior to observation also ensured time for the dam and litter to acclimate and negate any potential stress to new environment perception early on. Multiple cages were observed per day and the cage order was counterbalanced each day. The BORIS software was utilized during the observation time to track multiple behaviors, their frequencies, and duration. The research observer was blind to the litter ID and strain during litter observations. All litter observations were conducted during the light cycle between 7:00AM and 10:00AM.

Pregnant dams were checked daily for litter births, and the first day a litter was born was designated as P0. Litter observations were based on established literature showing that multiple brief observation period are sufficient to identify differences in rodent maternal care over preweaning development^17,30–32,32–34^. For cohort’s one and two, litter observations consisted of recording twelve snapshots of behavior conducted at 5-minute intervals over a one-hour period. Tally marks corresponding to observed behaviors were recorded during each spot checked. Measured dam behaviors observed included passive nursing, active nursing, licking and grooming, nest building, out of the nest, resting, running, and eating frequencies^17,31–34^. Passive nursing consisted of the dam resting with pups located ventral to the dam. Active nursing included pups located ventral to the dam while the dam was engaged in an active behavior such as licking and grooming, eating, drinking, autogrooming, and/or nest building. Passive and active nursing frequencies were also summed to analyze group differences in total nursing frequencies. Behavior of pups in each litter was also recorded and included pup crawling/running out of the nest, out of the nest, autogrooming, licking and grooming, eating solid food, nest building, jumping, digging, and climbing. Licking and grooming was recorded when a pup was observed to lick and groom the sibling or the dam. We were not able to differentiate individual pups in the litter, and therefore a behavior was recorded during each spot check if at least one pup in the litter displayed that behavior. In addition, the day at which developmental milestones were first observed were recorded for each strain and include eye opening, eating solid food, jumping, digging, and climbing.

For cohort three, the duration of litter behaviors and the frequency of nest exits were recorded for two offspring strain groups that were also observed in cohort two (B6-B6 and B6-CC12). During P4, P5, P11, P12, P18, and P19 observations for each cage took place over one hour and involved a researcher live scoring maternal behaviors for three 5-minute observation periods spaced fifteen minutes apart. Researchers were blind to the strain of the offspring while scoring. Maternal activity for three 5-minute epochs was then added together for each observation day and the total duration and frequencies of maternal care were analyzed across offspring strain groups.

### Statistical analyses:

Dam behavior was observed from all offspring strain groups for cohorts one and two for each day from P1–21 and the data are presented in the figures of dam behaviors across each day. Analyses that tested group differences in behaviors across P1–21 included average behavioral frequencies for litters that had at least 2 postnatal day observations during each postnatal week (PNW) to meet enough litters for statistical analyses^23^. Most litters had at least 3 observation days per PNW (see Figure 1B). Analyses that tested group differences in behaviors across PNWs included average frequencies of dam behaviors per litter for litters that had at least 3 postnatal day (PND) observations per PNW. The total number of observations, as well as the number of litters, included in the analyses of specific postnatal periods are listed in Figure 1. Tally marks totaling spot checks for each behavior and litter were summed for each postnatal day and used for data analysis for cohorts one and two. The average percent frequency of each behavior for each litter was then calculated across each time bin and analyzed for differences across offspring strain groups.

Stain differences in the percent of litters that survived to weaning were analyzed with Chi-squared tests. Strain differences in litter size, the percentage of males, and the percentage of *Chd8*^+/−^ offspring were analyzed with one-way ANOVA. Broad-sense heritability was estimated for B6-*Chd8*^+/−^ dam behaviors using one-way ANOVA for each trait to determine the proportion of variance accounted for by offspring strain compared with the total phenotypic variance. Principal component (PC) analyses were conducted on dams’ behavioral frequencies standardized trait values (z-scores) with a rotated component matrix, Varimax rotation, and Kaiser normalization. Regression scores were calculated based on each PC. Strain differences in dam and pup behaviors and principal component scores were analyzed with linear mixed effects models (LMM) with litter size, dam parity, the percentage of males, and the percentage of *Chd8*^+/−^ offspring per litter covaried as these variables can impact dam behaviors and the frequency pup behaviors were observed. For dam and pup behaviors that showed significant differences across offspring strain groups, post-hoc tests were Bonferroni corrected for multiple comparisons.

Spearman’s correlation coefficients were calculated to analyze relations between litter composition variables, dam behaviors, pup behaviors, adult behavioral traits, and adult Cohen’s D effect size estimates per strain previously reported^6^. Cohen’s D effect sizes were calculated by subtracting the mean trait values between groups and then dividing by the pooled standard deviation (SD). Cohen’s D effect sizes were calculated as follows (Mean1-Mean2)/√(((n1–1)*SD1^2^+(n2–1)*SD2^2^)/(n1+n2–2)). Decision tree regression analyses were conducted in Python with the scikit-learn package. Decision trees are valuable for understanding the interactions between independent variables and their collective impact on predicting a dependent variable, often providing greater accuracy compared to simple linear models. The model in this case is configured with a maximum depth of 5 and a minimum of 4 samples per leaf to balance complexity and generalizability. We then used SHAP (SHapley Additive exPlanations) interpretation to further understand the relationships in the decision tree models by quantifying the relative contribution of each litter observation variable to the *Chd8*^+/−^ effect size^26^. SHAP values interpret how individual variables influence the deviation from the expected value (average of the dependent variable) to the final predicted value, offering a transparent framework for interpreting a more complicated model such as a decision tree. The SHAP package in Python was used for generating SHAP values and graphs. Figures and graphs were constructed with SPSS, Python, Prism, and BioRender.

## Supplementary Material

1

## Data Availability

The data that support the findings of this study are openly available in Mendeley at (DOI here).
