# Supplementary material for: Offspring genetic diversity regulates rearing experiences that predict differential susceptibility to Chd8 haploinsufficiency
Source: Res Sq. 2025 Mar 3:rs.3.rs-6058389. Preprint. [Version 1] doi: 10.21203/rs.3.rs-6058389/v1 (PMC11908356; doi:10.21203/rs.3.rs-6058389/v1)
Supplement: 1 [file NIHPPrs6058389V1-supplement-1.pdf]

Supplementary Figures:

A. Number of litters observed per postnatal day (P) and strain

Strain	P1	P2	P3	P4	P5	P6	P7	P8	P9	P10	P11	P12	P13	P14	P15	P16	P17	P18	P19	P20	P21
B6-B6	19	12	8	9	7	6	11	16	12	8	8	9	10	13	16	13	7	8	6	8	12
B6-CC1	12	9	6	13	9	9	10	9	6	4	10	13	10	10	11	8	8	11	12	11	10
B6-CC10	7	6	6	6	4	6	7	8	7	6	7	6	8	7	9	7	5	6	7	9	5
B6-CC12	14	13	9	10	9	8	8	11	8	8	9	6	4	8	12	9	9	8	7	6	10
B6-CC13	5	3	4	3	3	7	5	5	4	4	3	3	7	6	5	5	2	7	4	6	7
B6-CC16	6	3	2	3	5	4	3	4	4	1	4	4	3	3	4	4	1	3	3	4	2
B6-CC2	3	4	4	6	6	4	4	3	5	4	5	4	5	5	2	4	5	6	4	5	4
B6-CC22	5	6	5	6	5	3	3	3	6	6	5	4	2	3	4	5	4	4	2	2	2
B6-CC24	6	5	3	2	5	5	6	7	6	4	3	5	5	7	7	3	6	3	5	6	7
B6-CC25	5	6	7	5	6	4	3	4	4	5	2	3	3	4	3	5	2	4	2	5	4
B6-CC28	6	3	4	4	5	5	5	4	4	3	5	5	5	4	5	5	3	6	4	5	4
B6-CC32	8	10	8	4	3	7	7	6	6	5	6	5	6	3	6	7	3	5	5	6	3
B6-CC44	4	3	5	5	6	6	5	5	2	6	4	6	6	5	6	2	4	5	5	5	5
B6-CC57	6	4	1	5	4	5	5	4	6	3	5	4	5	5	6	5	4	3	5	6	3
B6-CC7	8	6	8	5	4	5	4	5	2	3	3	3	5	5	3	4	1	5	3	5	5
B6-CC75	5	6	5	6	4	3	6	6	6	5	5	6	3	6	5	6	6	6	5	2	7

Max / Min

B.

Correlation Coefficients	Litter size	% Males	% *Chd8*$^{+/-}$
% Males	-0.11		
% *Chd8* Hets	**0.20**	0.02	
Nest quality	**-0.52**	**0.22**	-0.07
Passive nurse	**-0.32**	-0.08	-0.11
Active nurse	-0.05	-0.05	0.09
Lick & groom	**-0.20**	-0.02	-0.07
Nest build	-0.09	0.09	-0.04
Out of nest	**0.48**	-0.05	0.12
Eat	**0.48**	0.02	0.09
Rest	**-0.32**	-0.02	-0.11
Run	**0.22**	0.04	0.07
PC 1	**0.41**	0.00	0.16
PC 2	**-0.18**	-0.02	0.01

Correlation coefficient: + Max / - Max

C.

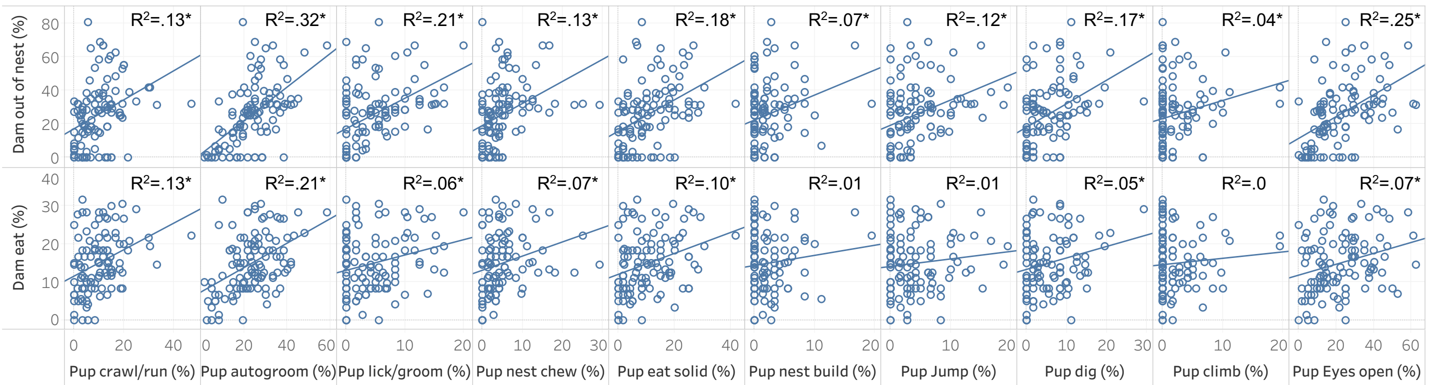

Supplementary Figure 1: (A) The number of litters observed for each offspring strain group are listed in the heatmap across each postnatal day (P). (B) The heatmap details Spearman's correlation between litter composition variables including litter size, the percentage of males per litter (% Males), and the percentage of *Chd8*$^{+/-}$ pups per litter with nest quality scores, dam behaviors, and dam principal component (PC) scores. Bold text indicates statistically significant correlations. (C) Scatter plots representing the correlations between dam behaviors, including dam out of the nest and dam eating percent frequencies, with pup activity percent frequencies. R^2 values reflect the coefficient of determination from univariate regression analyses and asterisks reflect statistically significant predictors.

		Litters excluded			
**Strain**	**Litters included in PNW 3 analyses**	**Eye opening**	**Eat solid food**	**Digging**	**Jumping**
**B6-B6**	14	0	2	8	8
**B6-CC1**	11	0	2	4	5
**B6-CC10**	6	0	0	1	6
**B6-CC12**	9	0	1	5	4
**B6-CC13**	7	0	2	1	1
**B6-CC16**	4	0	0	0	1
**B6-CC2**	6	0	0	1	1
**B6-CC22**	6	0	0	1	0
**B6-CC24**	7	0	0	1	1
**B6-CC25**	5	0	0	0	0
**B6-CC28**	6	1	1	1	1
**B6-CC32**	8	0	0	0	0
**B6-CC44**	6	0	0	1	0
**B6-CC57**	6	0	0	1	1
**B6-CC7**	5	0	1	0	0
**B6-CC75**	7	0	1	4	1

Supplementary Figure 2: The number of litters that were excluded from analyses for strain differences in the age to reach developmental milestones. Litters were excluded because they were not observed until postnatal day 21 or because they failed to display the behavior.

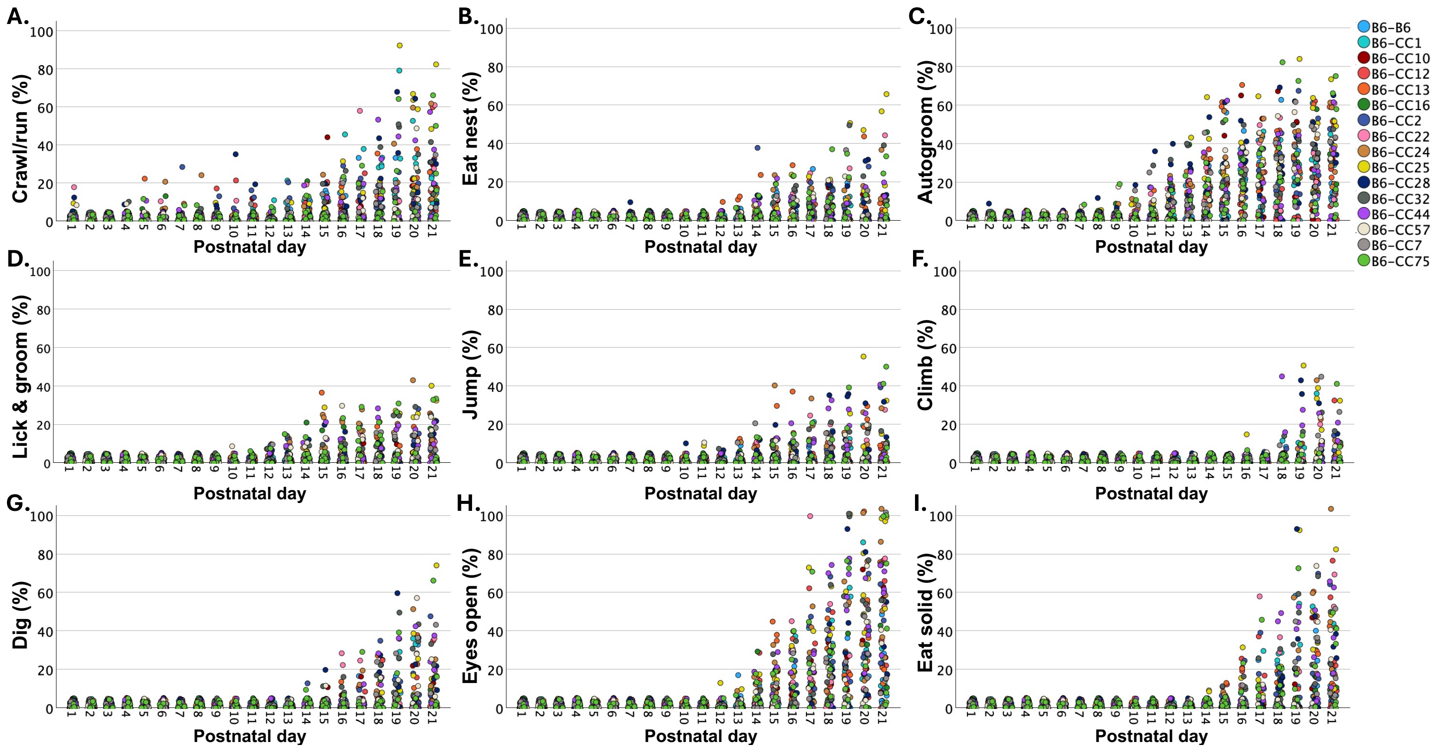

Supplementary Figure 3: (A-K) The frequency of behaviors observed across offspring strain groups from postnatal day 1-21 including the percent frequency of pup crawling/running (A), eating the nest (B),

autogrooming (C), licking and grooming (D), jumping (E) climbing (F), digging (G), eyes open (H), and eating solid food (I). Different colors reflect different strain groups that are detailed in the key.

Supplemental Tables:

Supplementary Table 1: Bonferroni post-hoc comparisons of differences in B6-*Chd8*$^{+/-}$ dam behavioral frequencies averaged between offspring strain groups during P1-21 and postnatal weeks 1-3. Strain means, standard deviations, and the number of litters per strain group are detailed. Cohen's D effect size estimates quantified the magnitude of differences between strains.

Supplementary Table 2: Bonferroni post-hoc comparisons of differences in pup behavioral frequencies across strains observed during P1-21 and postnatal week 3. Strain means, standard deviations, and the number of litters per strain group are detailed. Cohen's D effect size estimates quantified the magnitude of differences between strains.

Supplementary Table 3: Results from univariate regression analyses of significant predictors of postweaning traits. All strains were combined, and litter observation variables are averaged across postnatal day 1-21.

Supplementary Table 4: Heatmap of Spearman's correlation coefficients between litter compositions, dam and pup behaviors, and Cohen's D effect size estimates for trait disruptions between WT and *Chd8*$^{+/-}$ males and females for each strain. See Tabbaa, Knoll, and Levitt (2023) for details regarding behavioral tests and measurements. Litter composition variables include litter size, the percentage of males per litter, and the percentage of *Chd8*$^{+/-}$ pups litter. Dam behaviors measured including total nursing, passive nursing, active nursing, licking and grooming (L/G), nest building, out of the nest, eating, resting and running frequencies. Pup behaviors including autogrooming, licking and grooming, crawling/running, nest building, jumping, climbing, digging, eyes open, and eating solid food frequencies. All strains are combined, and statistically significant correlation coefficients are bolded.
